# Blastocyst Morphology in Oocyte Donors stimulated During the Late Follicular Phase with either rFSH:rLH or HP-hMG in a PPOS protocol: a Pilot Randomized Controlled Trial

**DOI:** 10.5935/1518-0557.20260030

**Published:** 2026

**Authors:** Fiorella Castillo-Velásquez, Silvia Ortiz, Paula Ricra, Camila Carlos, Luis Noriega-Portella, Juan-Enrique Schwarze, Luis Noriega-Hoces, Pamela Villanueva, Luis Guzmán

**Affiliations:** 1 Grupo PRANOR, Lima, Peru; 2 Clínica Concebir, Lima, Peru; 3 Merck Healthcare KGaA, Darmstadt, Germany

**Keywords:** oocyte donor, progesterone-primed ovarian stimulation, PPOS, r-hFSH:r-hLH, HP-hMG

## Abstract

**Objective:**

The aim of this study was to investigate whether there were differences in the number of good-quality blastocysts after 6 days of treatment with r-hFSH followed by late follicular phase treatment with r-hFSH:r-hLH in a 2:1 fixed ratio combination or HP-hMG in a PPOS protocol in a population of young women.

**Methods:**

Healthy oocyte donors were randomized to Group 1 or Group 2 (40 women per group). All donors were treated with 200 mg/day oral progesterone from Day 1 and 225 IU/day r-hFSH for the first six days of ovarian stimulation. From Day 7 onwards, Group 1 received 225 IU/day r-hFHS:r-hLH 2:1 fixed ratio combination and Group 2 received 225 IU/day HP-hMG. Insemination was performed using intracytoplasmic sperm injection. Embryo quality was graded using the SART classification. The primary outcome was the number of blastocysts with good morphology (AA or AB).

**Results:**

The mean (SD) number of good-quality blastocysts was significantly higher in Group 1 compared with Group 2 (3.4 [2.0] vs. 2.3 [2.0]; difference 0.9 (0.6 to 1.7); Kruskal-Wallis test p=0.0359). There was no difference in total gonadotropin dose, length of stimulation or number of oocytes retrieved between the groups.

**Conclusion:**

Treatment with r-hFSH:r-hLH in a 2:1 fixed ratio combination starting in the late follicular phase resulted in a significantly higher number of good-quality blastocysts compared with HP-hMG in oocyte donors receiving PPOS.

## INTRODUCTION

In recent years, declining fertility rates has been observed worldwide; for example, in the USA, a 4% decrease in the number of live births was recorded between 2019 and 2020 (Eisenberg *et al*., 2021). Globally, the reasons for this decline are multifactorial, although delayed childbirth due to personal, professional or financial reasons may be a key contributing factor in developed countries (Mertes & Pennings, 2011). However, women who delay childbirth for whatever reason may face problems conceiving, owing to the age-related decline in fertility, as advanced maternal age (AMA) can impact oocyte and embryo competence through several mechanisms, with 35 years defined as the lowest age threshold to define AMA (Cimadomo *et al*., 2018).

Assisted reproductive technology (ART) may be an option for women who delay childbirth to achieve their desire to build a family; however, these women may still have a high chance of recurrent failed cycles (Bashiri *et al*., 2018) or low prognosis for live birth (Schmidt *et al*., 2012). In some cases, oocyte donation from healthy, fertile women who voluntarily undergo ovarian stimulation (OS) is an option for women who delay childbirth (Lutjen *et al*., 1984; Hammoud *et al*., 2009). While the legality and legislative framework for oocyte donation varies considerably among countries (Calhaz-Jorge *et al*., 2020; López *et al*., 2021), current indications for women seeking oocyte donation include hypergonadotropic hypogonadism, diminished ovarian reserve, having (or being a known or suspected carrier of) a significant genetic defect or a family history of a condition, poor oocyte and/or embryo quality or multiple previous failed attempts to conceive via ART. In addition, where permitted, oocyte donation may be an option for men who do not have a female partner or those who have a trans-female partner and are planning to use a gestational carrier (Lutjen *et al*., 1984). AMA is also an indication for women seeking oocyte donation, usually due to delayed childbirth and/or in conjunction with the above-mentioned indications (Cimadomo *et al*., 2018).

Several exogenous gonadotropin preparations are indicated for OS, including highly purified human menopausal gonadotropin (HP-hMG), which is purified from the urine of postmenopausal women and comprises FSH and LH-like activity derived from human chorionic gonadotropin (hCG), and recombinant human FSH (r-FSH) and LH (r-LH), which are produced using recombinant DNA technology (Lunenfeld *et al*., 2019). The recombinant products are available as separate products (EMA, 2022a; EMA, 2022b) or as a 2:1 fixed-ratio combination product (EMA, 2023). In the general population undergoing ART procedures, OS with r-hFSH:r-hLH 2:1 has been reported to yield a higher number of oocytes retrieved than HP-hMG (Pacchiarotti *et al*., 2010; Fábregues *et al*., 2013; Revelli *et al*., 2015; Kirshenbaum *et al*., 2021). However, there is little published evidence on oocyte quantity or oocyte quality following any specific treatment protocol in the context of oocyte donors. A recent systematic review and meta-analysis identified five studies that evaluated the effect of the type of gonadotropin used for OS in oocyte donors (Martinez *et al*., 2021). Three of these studies compared HP-FSH *versus* hMG (Söderström-Anttila *et al*., 1996), r-hFSH *versus* r-hFSH + hMG (Tesarik *et al*., 2002) or r-hFSH *versus* HP-hMG *versus* r-hFSH + HP-hMG (Melo *et al*., 2010), respectively, in a long agonist protocol; and two studies compared r-hFSH *versus* r-hFSH + r-hLH (Acevedo *et al*., 2006) or corifollitropin alfa *versus* hMG (Cruz *et al*., 2017), respectively, in a gonadotropin releasing hormone (GnRH) antagonist protocol. Although no difference in the number of oocytes retrieved was reported between the studies using GnRH antagonists versus agonists, no further conclusions could be drawn owing to the high degree of heterogeneity among the studies.

As an alternative to using GnRH analogs to prevent the premature luteinizing hormone (LH) surge during OS, recent work has focussed on the use of stimulation cycles using progesterone (progestin-primed OS [PPOS]) to prevent early luteinisation (La Marca & Capuzzo, 2019; Glujovsky *et al*., 2023). PPOS protocols are advantageous in that they can be administered orally, are flexible and economic for patients, and control over the LH surge is achieved rapidly (Kuang *et al*. 2015; Qin *et al*., 2016, Massin, 2017). These factors highlight the potential of PPOS to be a cost-effective and patient-friendly oral alternative to injected GnRH analogs in younger women, whose motivation for oocyte donation is usually solely altruistic and, therefore, presents unique challenges for physicians when balancing the risk and benefits to ensure the wellbeing and convenience of the donor (Kool *et al*., 2018).

Recent studies have demonstrated few or no differences between PPOS and a GnRH antagonist protocols in oocyte quality (Yildiz *et al*., 2019) or competence (Vaiarelli *et al*., 2024), oocyte and MII oocyte number and number of cleavage and blastocyst embryos (Duc Thang *et al*., 2024), oocyte maturation rates and trigger-to-oocyte retrieval interval (Ata *et al*., 2024). However, there is no consensus on which populations of women may most benefit from PPOS (e.g., poor responders, women with endometriosis, women of advanced maternal age, young women for oocyte donation procedures) (Glujovsky *et al*., 2023). Furthermore, there are no robust data on any potential interaction between supraphysiological levels of progesterone in PPOS and the different LH-like activity in the commonly used preparations, and the effect this may have on oocyte quality.

The objective of the present study was to investigate the number of good-quality blastocysts obtained from population of young women treated in the late follicular phase with r-FSH:r-LH in a 2:1 fixed ratio combination or HP-hMG, following 6 days of treatment with r-FSH in a PPOS protocol in a population of young women.

## MATERIALS AND METHODS

### Study design

**Figure 1:**
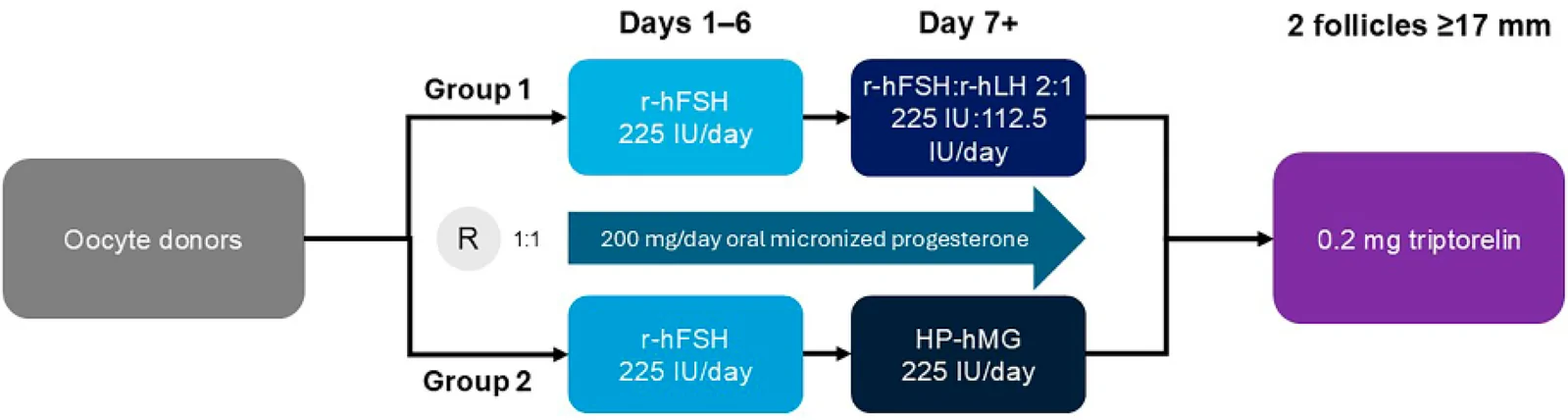
Study design.

The aim of this pilot randomized controlled trial was to compare the blastocyst morphology of donor oocytes following OS with r-FSH:r-LH in a 2:1 fixed ratio combination (Pergoveris; Merck KGaA, Darmstadt, Germany) or HP-hMG (Menopur; Ferring, West Drayton, UK), both starting from stimulation Day 7 following 6 days of stimulation with r-FSH alfa (Gonal-f; Merck KGaA, Darmstadt, Germany). The study was conducted from August 2022 to June 2023 at a single centre in Lima, Peru. The study design is shown in Figure 1.

### Eligibility of gamete donors

Women undergoing OS for oocyte donation were screened under informed consent for inclusion and exclusion criteria through blood testing, and general medical evaluation.

Women were eligible as oocyte donors if they met all the following criteria: age 19-30 years at the time of providing informed consent; recorded levels of AMH ≥2 ng/mL, and BMI 21.0-29.9 kg/m^2^. All donors underwent physical, gynecological and psychological examinations. Additionally, they should be no family history of hereditary diseases or chromosomal disorders. All participants had a normal karyotype, and tested negative for sexually transmitted diseases and toxicological screening, and had an up-to-date negative cervical cancer screening. Recruitment of oocyte donors was done based on recommendations given by other donors and the reason for gamete donation was solely altruistic.

Sperm was obtained from the partners of the oocyte recipients (homologous) or from sperm donors (heterologous) in the case of poor semen quality (Boitrelle *et al*., 2021). Men aged 21-50 years were eligible as sperm donors if they met the criteria for donation at the treating clinic or another certified cryobank. Sperm quality was assessed according to semen volume, and sperm motility, quantity and morphology.

### Randomization

Participants were randomized (1:1) using a computer-generated algorithm to either Group 1 (r-hFSH:r-hLH 2:1 fixed ratio combination) or Group 2 (HP-hMG) before receiving the first injection for OS. No blinding was performed.

### Interventions

All donors were stimulated with 225 IU/day r-FSH (Gonal-f, Merck KGaA, Darmstadt, Germany) for the first six days. All donors received 200 mg/day oral micronized progesterone from Day 1 of stimulation (PPOS). From Day 7 onwards, oocyte donors in Group 1 were treated with 225 IU/day r-hFHS:r-hLH 2:1 fixed ratio combination (150 IU r-FSH: 75 IU r-hLH), and oocyte donors in Group 2 were treated with 225 IU/day HP-hMG (Figure 1).

Follicular development was monitored by serial ultrasound scans. The first scan was conducted at the beginning of the cycle, with the second scan after 5 days of gonadotropin treatment, and then every 2 days until follicle size reached 16 mm; a final scan was conducted on the day of ovarian maturation trigger, when two follicles had reached a mean diameter ≥17 mm. Oocyte maturation was triggered using 0.2 mg triptorelin, with oocyte pick-up at 34-36 hours after triggering.

### Insemination

Homologous sperm was obtained from the recipient’s partner in accordance with clinic protocols. Heterologous sperm was collected from the cryobank local to the treating centre or from another certified cryobank, and then transferred to the clinic as a frozen specimen in accordance with clinic standards. All inseminations were performed using intracytoplasmic sperm injection (ICSI).

### Embryo culture and implantation

Zygotes were cultured individually until day 5/6 in drops of 25 µL of Global Total® media (Lifeglobal, Canada) under mineral oil at 37 °C in 7% CO_2_, 5% O_2_ and 88% N_2_ (adapted from Sepúlveda *et al*., 2009). Embryos were classified according to the Society for Assisted Reproductive Technology (SART) classification (Racowsky *et al*., 2010).

### Embryo vitrification

Embryos were vitrified using a modified Cryotop method (Kuwayama *et al*., 2005). Embryos were equilibrated at room temperature for 12 minutes in the equilibrium solution (TCM-199 medium/20% synthetic serum substitute/7.5% (v/v) ethylene glycol/7.5% dimethyl sulfoxide). Embryos were then transferred to the vitrification solution (TCM-199 medium/20% synthetic serum substitute/15% ethylene glycol/15% dimethyl sulfoxide/0.5 M sucrose) and incubated for 1 minute before being placed in a cryoslip (one embryo per strip) and immediately submerged in liquid nitrogen.

### Outcomes

#### Primary outcome

The primary outcome was the number of blastocysts with good morphology. All embryos were evaluated by two senior embryologists using the SART classification (Racowsky *et al*., 2010) and categorized as either good, fair or poor: a good grading was assigned to embryos with Grade A inner cell mass and trophectoderm Grade A or B (AA or AB blastocysts); a fair grading was assigned to embryos with Grade B inner cell mass and trophectoderm Grade A, B or C (BB, BC or BA blastocysts); a poor grading was assigned to embryos with Grade C inner cell mass (CC or CB blastocysts).

#### Secondary outcomes

Secondary outcomes were the total number of oocytes recovered and inseminated, the number of 2PN embryos, the total number of blastocysts, and the proportion of good-quality blastocysts, calculated as the proportion of embryos to be of good quality over all embryos assessed.

#### Data collection/monitoring

All donors were monitored for adverse events during OS, and were asked to self-report any adverse events occurring within two weeks after oocyte retrieval. The potential risks associated with the study were those common to all ART procedures: the risks of OS were ovarian hyperstimulation syndrome (OHSS) and ovarian torsion; the risks of egg retrieval were bleeding, risks associated with anaesthesia, infection, and trauma.

Monitoring involved regular transvaginal ultrasound scans and hormonal blood tests to monitor follicular development and ovarian response. Ultrasound scans were performed as previously described. Donors were scheduled for a follow-up clinical evaluation throughout the two weeks after oocyte retrieval to assess recovery and detect any delayed adverse events. Any adverse events reported by donors were to be documented in real time by the clinical team and coded using the Medical Dictionary for Regulatory Activities (MedDRA). The data collection process adhered to established clinical protocols and ethical guidelines, ensuring donor safety and compliance with research standards.

### Statistical analysis

We estimated that 80 participants would be required to obtain 80% power to demonstrate a difference of at least one blastocyst in the number of good-morphology blastocysts, assuming a mean number of blastocysts in the HP-hMG group of 4.9, based on our previous observations and assuming a loss rate of 10%. Between-group differences in blastocyst morphology were assessed using the Kruskal-Wallis test. All other analyses were descriptive.

### Ethical approval

This study protocol was approved by the local Ethics Committee. All participants provided informed consent after counselling. Consent was obtained by the investigator or a member of their team directly from the participants before that subject takes part in the study. Consent was documented either with a handwritten signature on paper or through an electronic signature following the Guidance on Use of Electronic Informed Consent, according to the patient’s preference. All participants received a copy of the Informed Consent Form.

## RESULTS

### Participants

#### Oocyte donors

Of the 80 donors recruited, seventy-nine were included in the analysis. One donor in Group 1 was excluded after their OS was cancelled. The baseline characteristics were similar between the women randomly assigned to either group (Table 1).

**Table 1:** Baseline characteristics and stimulation results for the oocyte donors.

	Group 1 r-hFSH:r-hLH (2:1) (n=39)	Group 2 HP-hMG (n=40)
Age, years	24.0 (2.6) 18-30	24.2 (2.9) 19-29
AMH, ng/mL	4.3 (1.7) 2.1-11.8	4.5 (2.1) 2.3-11.8
BMI, k/m^2^	22.6 (18.9) 18.9-26.3	22.7 (2.1) 18.7-25.7
Weight, kg	58.9 (6.9) 46.0-77.5	58.7 (7.8) 45-77
Height, m	1.6 (0.1) 1.52-1.77	1.6 (0.1) 1.52-1.77
TSH, mIU/L	1.8 (0.9) 0.8-4.5	1.7 (0.7) 0.8-4.0
Prolactin, ng/mL	15.6 (7.3) 1.9-29.9	15.6 (6.3) 6.9-29.6
Total dose, IU	2140 (205) 1800-2700	2120 (227) 1800-3150
Length of stimulation, days	9.5 (0.9) 8-12	9.4 (1.0) 8-14
Oocytes recovered	22.8 (10.4) 9-57	22.4 (10.3) 6-51

Data presented as mean (SD), min-max.

AMH, Anti-Müllerian Hormone. BMI, body mass index. HP-hMG, highly purified human menopausal gonadotropin. IU, international units. SD, standard deviation. r-hFSH, recombinant human follicle stimulating hormone, r-hLH, recombinant human luteinising hormone. TSH, thyroid stimulating hormone.

### Sperm donors

The mean paternal age was 36 (SD 10) years in Group 1 and 37 (SD 12) years in Group 2. Most semen samples were homologous (Group 1: 72.0%; Group 2: 27.7%) compared with heterologous (Group 1: 28.8%; Group 2: 27.8%) and the highest proportion of inseminations used fresh semen (Group 1: 48.4%; Group 2: 53.3%) rather than frozen semen (Group 1: 51.6%; Group 2: 46.7%).

### Ovarian stimulation

The total FSH dose was 2140 (SD 205) IU in Group 1 and 2120 (SD 227) IU in Group 2. The length of stimulation was 9.5 (SD 0.9) days in Group 1 and 9.4 (SD 1.0) days in Group 2 (Table 1). The mean number of oocytes retrieved was 22.8 (SD 10.4) in Group 1 and 22.4 (SD 10.3) in Group 2 (Table 1).

### Outcomes

#### Primary outcome

Overall, 97 embryo transfers were registered, 50 in Group A and 47 in Group B. The mean (SD) number of good-quality blastocysts was statistically significantly higher in the women in Group 1 compared with those in Group 2 (3.4 [2.0] *vs*. 2.3 [2.0]; difference 0.9 [95% CI 0.6 to 1.7]; Kruskal-Wallis test *p*=0.0359) (Table 2). There were no differences in the number of fair-quality (0.4 [95%CI -0.18 to 1.0]) or poor-quality (0.5 [95% CI -0.4 to 0.3]) blastocysts between the treatment groups.

**Table 2:** Results of ovarian stimulation per stimulation cycle following ICSI

	Group 1 r-hFSH:r-hLH 2:1 (n=50)	Group 2 HP-hMG (n=47)	Difference Group 1- Group2 (95% CI)
Oocytes inseminated	11.3 (1.4) 7-13	11.6 (1.9) 6-14	-0.3 (-1.0 to 0.4 )
Semen donor (heterologous/homologous), n (%)	14/36 (28.0)	13/34 (27.7)	0.3 (-23.5 to 22.2)
2PN embryos	8.3 (2.3) 3-13	8.5 (2.0) 4-12	-0.20 (-1.1 to 0.7)
Total blastocysts	6.1 (6.1) 2-11	4.8 (2.5) 0-10	1.4 (0.3 to 2.4)
Number of blastocysts with Good morphology^*^ (per recipient)	3.4 (2.0) 0-9	2.5 (2.0) 0-7	0.9 (0.6 to 1.7)†
Proportion of blastocysts with Good morphology^*^, %	55.3	47.1	8.2 (-20.0 to 25.6)
Number of blastocysts with Fair morphology^*^	2.0 (1.5) 0-5	1.6 (1.3) 0-4	0.4 (-0.18 to 0.1)
Number of blastocysts with Poor morphology^*^	0.8 (1.0) 0-4	0.9 (0.9) 0-3	-0.04 (-0.4 to 0.3)

Results are presented as mean (SD), min-max, unless stated otherwise. Some participants underwent more than one stimulation cycle; therefore, the number of cycles is higher than the number of donors.

* Blastocyst morphology assessed according to the Gardner and SART grading systems.

†Kruskal-Wallis test *p* value = 0.0359.

AMA, advanced maternal age. BMI, body mass index. HP-hMG, highly purified human menopausal gonadotropin. POR, poor ovarian reserve. r-hFSH, recombinant human follicle stimulating hormone, r-hLH, recombinant human luteinising hormone.

#### Secondary outcomes

Group 1 had a higher total number of blastocysts compared with Group 2 (difference 1.4 [95% CI 0.3 to 2.4]) (Table 2). There were no differences in the total number of oocytes inseminated (difference 0.3 [95% CI -0.4 to 1.0]), the total number of 2PN embryos (difference 0.2 [95% CI -0.7 to 1.1]), or the proportion of good-quality blastocysts (difference 8.2% [95% CI -20.0% to 25.6%]) between treatment groups (Table 2). Finally, ongoing pregnancy rates were similar among the group (62.5% for Group 1 and 58.9% for Group).

### Safety

No adverse events were identified during the study. There were no reports of OHSS, ovarian torsion or other procedure-related complications.

## DISCUSSION

The results of this randomized pilot study show that OS with r-FSH:r-LH in a 2:1 fixed ratio combination from Day 7 resulted in a significantly higher number of good-quality blastocysts compared with HP-hMG from Day 7 in oocyte donors using a PPOS protocol to prevent premature LH surge. These preliminary results using PPOS in healthy, young women show a potentially lower oocyte developmental competence when urinary gonadotrophins are used in combination with follicular phase progesterone in this population.

Premature LH surge can compromise oocyte yield in *in*-*vitro* fertilization (IVF)/ICSI and is one of the major reasons for cycle cancellation in OS (Messinis, 2006). However, the use of GnRH analogs (either GnRH agonists or GnRH antagonists (Macklon *et al*., 2006)) have recognised drawbacks. GnRH agonists increase the risk of OHSS through the requirement for hCG triggering, whereas GnRH antagonists can have variable effectiveness in some patient populations, including older patients and those with diminished ovarian reserve (Bosch *et al*., 2003; Reichman *et al*., 2014). Furthermore, both GnRH agonists and antagonists are injectable products, which increases the cost, complexity and patient burden of OS.

Studies using progesterone in the follicular phase have shown that it is a regulator of ovulation timing and a potentially convenient and flexible oral alternative to GnRH agonists for preventing early luteinization when used in a PPOS protocol in freeze-all cycles (Kuang *et al*., 2015). Most of the studies on PPOS published to date have compared the outcomes GnRH analog protocols and PPOS using locally available preparations of hMG for OS, although there are a few studies that directly compare the outcomes from different gonadotropin preparations. In a retrospective study covering two discrete time periods (2019 and 2020), during which two different OS regimens were used (2019: corifollitropin, recombinant FSH and GnRH antagonists, with ovulation triggered using GnRH agonist; 2020: PPOS protocol with human menopausal gonadotropin [hMG] and dual trigger [GnRH agonist and low dose hCG]), there was no difference in the ratio of the number of mature oocytes and serum AMH between the two periods (4.0 [2.3-7.1] *vs*. 4.0 [2.7-6.8]; *p*=0.80), although the total cost of drugs used for OS was lower in the second period €940 [€774-€1,096] *vs*. €520 [€434-€564]; *p*<0.001) (Filippi *et al*., 2023). In addition, a retrospective study in women who were stimulated with r-FSH (Gonal-f, Merck KGaA, Germany) or HP-hMG (Merional, IBSA Institut, Switzerland) in a PPOS protocol (Dydrogesterone [Duphaston® 10 mg, Abbott, Turkey]) reported that the total number of follicles, oocytes, MII and 2PN oocytes, fertilization rates and pregnancy rates were similar between the two groups, although higher doses of gonadotropin were used in the HP-hMG group compared with the r-FSH group (*p*=0.001) (Yetkinel *et al*., 2024). While these early results may demonstrate the promise of using PPOS cycles, they do not address any potential differences in the gonadotropin preparations that are commonly used in OS, specifically the FSH and LH activity in r-FSH:r-hLH 2:1, which are produced using complementary DNA technology and have a high level of purity and batch-to-batch consistency (Bassett & Driebergen, 2005; Lispi *et al*., 2006; Bassett *et al*., 2009; Lispi *et al*., 2023) or the LH-like activity in HP-hMG, which is mainly derived from hCG purified from pregnant women (Capolupo *et al*., 2024). Furthermore, there are no studies that investigate how these components may interact with the supraphysiological concentrations of progesterone used in PPOS.

Although LH and hCG share approximately 85% structural identity and bind to the same receptor (LHCGR) on the antral follicles, they cannot be considered equivalent, as they interact differently with the receptor and activate different cellular pathways (Casarini *et al*., 2018). The action of LH is preferentially exerted through phosphorylated extracellular-regulated kinase 1/2 (pERK1/2) and phosphorylated AKT (also known as protein kinase B), resulting in proliferative/antiapoptotic signals and partial agonism of progesterone production *in vitro*. In contrast, hCG displays notable cAMP/protein kinase A (PKA)-mediated steroidogenic and proapoptotic potential (Casarini *et al*., 2018). Therefore, on the one hand, r-hLH may have a positive effect on oocyte maturation, potentiated by r-FSH, as well as having an anti-apoptotic effect on cumulus cells and promoting the paracrine signalling required for cell expansion and oocyte signalling during folliculogenesis (Ruvolo *et al*., 2007; Huang *et al*., 2015). On the other hand, the hCG in HP-hMG may have a detrimental effect on oocyte development by activating the steroidogenic and proapoptotic pathways.

Progesterone contributes to normal mammalian ovarian function and has several critical functions during embryo development and implantation, including endometrial receptivity, embryonic survival during gestation, and transformation of the endometrial stromal cells to decidual cells (Salehnia & Zavareh, 2013). While hCG has greater steroidogenic potential than LH, reflecting its role during pregnancy to support increased progesterone production, the dual proliferative and androgen-stimulating roles exerted by LH in granulosa and theca cells, respectively, during folliculogenesis might not require induction of the steroidogenic pathway at the levels needed in pregnancy (Casarini *et al*., 2018). Furthermore, high levels of progesterone, such as the supraphysiological levels due to PPOS, may even have local doseand species-dependent effects on the developing oocyte (diZerega & Hodgen, 1982; Kim & Greenwald, 1987; Setty & Mills, 1987; Komatsu & Masubuchi, 2017). In a retrospective analysis of 1200 cycles of women stimulated with hMG in either a PPOS or GnRH antagonist protocol, at similar cumulative hMG doses, the number of follicles with diameter >10mm, the number of retrieved oocytes and the number of MII oocytes were lower in the PPOS protocol compared with the antagonist protocol, although the oocyte yields were rescued by higher doses of hMG (Long *et al*., 2021). These exploratory results we report here add weight to these preliminary data on the potential detrimental effect of the LH-like activity of hCG in conjunction with high levels of progesterone from PPOS, although this needs further investigation.

While we report a higher number of good-quality blastocysts, we did not observe a difference in the number of oocytes retrieved, which is contrary to the results of other studies that compare these two formulations. In a meta-analysis of six studies of OS with r-FSH:r-hLH 2:1 fixed ratio combination versus HP-hMG, a higher mean number of oocytes was retrieved in the rFSH:r-LH 2:1 fixed ratio combination cohort (1.43 [95% CI 0.18, 2.69]; *p*=0.025) (Dahan *et al*., 2026). However, the studies analysed reported on women undergoing routine IVF cycles using autologous oocytes, and there are few published data on the use of these treatments in donor cycles. In one study of donor cycles, treatment with r-FSH:r-hLH in a 2:1 ratio also resulted in a higher number of oocytes retrieved (16.5 [SD 4.1] *vs*. 11.8 [SD 2.6]; *p*=0.049) compared with treatment with urinary gonadotropins (HP-hMG plus uFSH) (Requena *et al*., 2014). This variance from the published literature warrants further investigation into the outcomes of different OS protocols specifically in donor cycles.

The strengths of this trial are it was a randomized pilot trial conducted at a single centre that recruited a representative population of women participating in oocyte donation. Some limitations also have to be acknowledged. Randomized treatment only commenced after stimulation Day 6, prior to which both cohorts were treated with r-FSH; therefore, the effect of the test compounds from Day 1 of stimulation could not be addressed, and no sperm DNA fragmentation was performed, also comparing the outcome to stimulation with rFSH alone.

## CONCLUSIONS

In this randomized controlled pilot study we show that late follicular phase treatment with r-FSH:r-LH in a 2:1 fixed ratio combination resulted in a significantly higher number of good-quality blastocysts compared with late follicular phase HP-hMG following OS with r-FSH until Day 6 in oocyte donors. While these data need to be confirmed in larger clinical studies, optimizing oocyte quality in PPOS cycles may improve the chances of live birth resulting from a single cycle, thus reducing the burden on oocyte donors.
